# Biomedical applications of polyelectrolyte coated spherical gold nanoparticles

**DOI:** 10.1186/s40580-019-0183-4

**Published:** 2019-04-24

**Authors:** Melanie A. Fuller, Ingo Köper

**Affiliations:** Flinders Institute for NanoScale Science and Technology, Flinders University, Bedford Park, SA 5042 Australia

**Keywords:** Polyelectrolyte, Gold nanoparticles, Nanoparticles, Gold, Biomedical

## Abstract

Surface modified gold nanoparticles are becoming more and more popular for use in biomaterials due to the possibility for specific targeting and increased biocompatibility. This review provides a summary of the recent literature surrounding polyelectrolyte coatings on spherical gold nanoparticles and their potential biomedical applications. The synthesis and layer-by layer coating approach are briefly discussed together with common characterisation methods. The potential applications and recent developments in drug delivery, gene therapy, photothermal therapy and imaging are summarized as well as the effects on cellular uptake and toxicity. Finally, the future outlook for polyelectrolyte coated gold nanoparticles is explored, focusing on their use in biomedicine. 
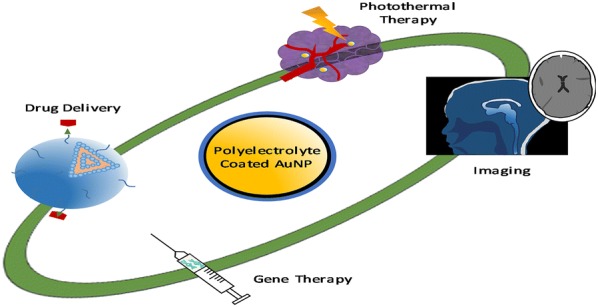

## Introduction

Gold nanoparticles (AuNPs) can be described as solid gold particles with a diameter between 1 and 100 nm, and have the potential to be used in a range of biomedical applications due to their unique physical and optical properties [1–4]. Examples of these unique properties include the surface plasmon resonance (SPR) effect, which can give information about the local particle environment as well as the physical dimensions of the particles. Furthermore, by employing the Surface Enhanced Raman Scattering (SERS), AuNPs can be used as probes to enhance Raman scattering applications [5–8]. After a typical synthesis, AuNPs are coated with an organic material or capping agent which provides stability to the particles. One of the advantages of AuNPs is that they can be easily functionalized with a range of different materials including antibodies, proteins, ligands, DNA, polymers and polyelectrolytes [9–11]. This ease of functionality is useful in many of the biomedical applications which are discussed here.

**Fig. 1:**
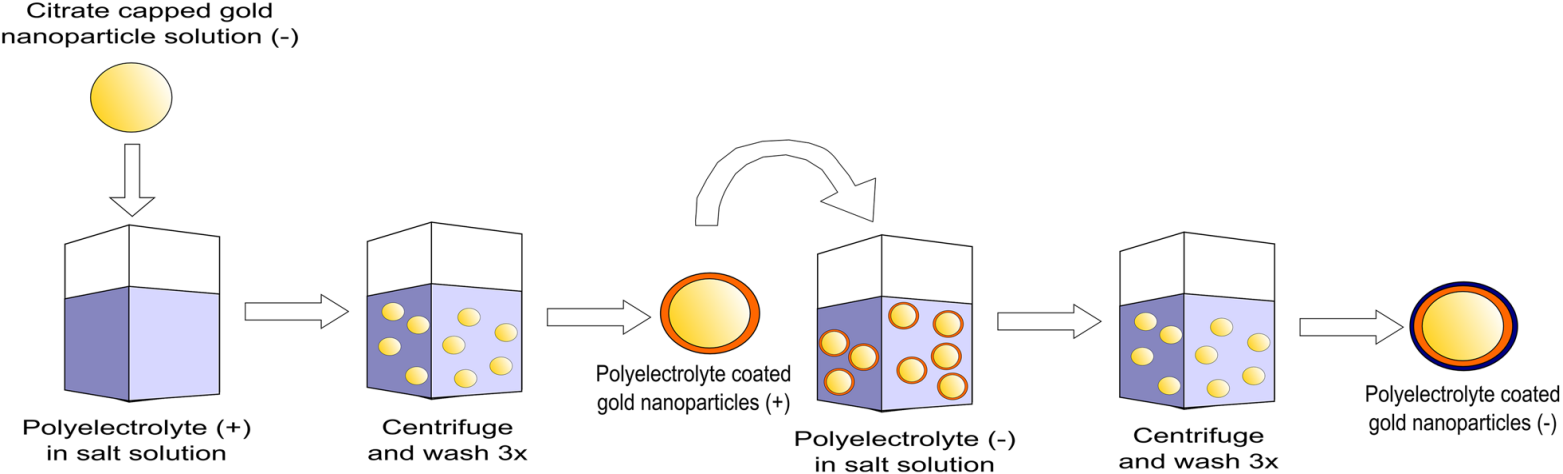
Method for self-assembly of polyelectrolyte-coated citrate capped gold nanoparticles using the Lbl method

Polyelectrolytes (PEs) are polymers of repeating units which contain an ionizable group [12]. These charged polymers can be used to coat surfaces and particles in a number of ways including covalent attachment, hydrogen bonding and electrostatic interactions between layers [13, 14]. While there are many ways PEs can be attached to nanoparticles (NPs), this review will focus on the electrostatic attachment of PE coatings as they are easy to produce and have a range of applications from microfluidics to water membrane filtration systems [14, 15]. Specifically, the Layer-by-layer (Lbl) approach will be reviewed, where PEs can be attached to a surface in a single or multilayer deposition. Essentially the Lbl electrostatic approach uses two solutions of opposite charge. A substrate can be dipped into a solution or a particle solution can be mixed with PEs to coat a surface (Fig. 1) [16, 17]. Once a coating of the polyelectrolyte has been added, the charge on the substrate or particle is inverted, and hence a subsequent polymer layer of opposite charge can be applied. The number of layers applied determines the total thickness of the polymer coating [13]. Due to the ease of deposition, Lbl polyelectrolyte coatings have been initially investigated on flat substrates for a wide range of applications, but now the coating of 3-dimensional objects is also being explored [15, 18, 19]. These objects, such as spherical nanoparticles, offer larger surface area to volume ratios and larger reactive surface areas, which is essential in drug delivery and catalysis [20, 21].

The coating efficiency of the PEs on NPs is influenced by the shape of the nanoparticle, the type, length and concentration of the polymer, as well as the total salt concentration in the solution used [22, 23]. Gold nanorods (GNRs) have been the most widely studied 3D particle shape for PE coatings, as their structure allows for relatively homogeneous coatings. Additionally, they have been extensively used in sensing applications, as their surface plasmon is particularly sensitive to changes in the local environment [20, 24–27]. This review however is focused on exploring spherical AuNPs, and comprehensive reviews of AuNRs can be found elsewhere [28, 29]. The curved surface of small (< 50 nm) NPs makes it difficult to form complete and homogeneous coatings, in part due to the lack of flexibility of the polymer chains, resulting in poor coverage on the nanoparticle surface and a decrease in stability of the coating [30]. In general, incomplete coatings can cause particle aggregation [23, 31, 32]. To avoid incomplete coatings, the polymer which is coating the NP needs to be in excess to ensure complete coverage on the surface [23]. Similarly the addition of salt in the coating solution typically allows for more flexibility in the PE chains, which can lead to an improved coverage of the particles [22, 33–35].

## Synthesis of polyelectrolyte coated nanoparticles

There are many different approaches to synthesizing AuNPs, each aiming to control nanoparticle size, shape and surface functionality [36–38]. In the Turkevich method, hydrogen tetrachloroaurate (HAuCl_4_) is treated with citric acid in boiling water, with the citrate acting both as a reducing and stabilizing agent [39, 40]. This method produces NPs with diameters in the range of 10–20 nm, with the particle size being controlled by the gold to citrate ratio [41]. Alkanethiol-stabilized AuNPs, which are soluble in organic solvents, can be formed using tetraoctylammonium bromide (TOAB) as the capping agent and sodium borohydrate (NaBH_4_) as the reducing agent [42]. Depending on the gold-to-thiol ratio, temperature and reduction rate, NPs between 1.5 and 5 nm in diameter can be produced [42]. Other synthesis methods resulting in size and particle uniformity distributions use different reducing agents such as sucrose, ethylenediaminetetraacetic acid, fruit extracts and amines [43–47].

The capping agent used in the NP synthesis influences which PE can be used for the initial coating. The Lbl technique is based on the attraction of oppositely charged layers and is the main interaction employed in coating AuNPs with PEs. Commonly used PE polymers are shown in Table 1. The charge of the PE is important as the polymer will electrostatically attach to a particle only if the capping agent is of opposite charge [14]. For example, after the Turkevich method which caps the particles in negatively charged citrate, only positively charged PEs will attach.

**Table 1:** Commonly used polyelectrolytes showing their charge at pH 7 and their current applications

Abbreviation	Name	Charge at neutral pH	Application	References
PEI	Polyethyleneimine	Positive	Gene therapy, drug delivery	[48, 49]
PAH	Polyallylamine hydrochloride	Positive	Drug delivery, gene therapy	[50, 51]
PSS	Polystyrene sulfonate	Negative	Drug delivery	[52, 53]
PLL	Poly-l-lysine	Positive	Gene therapy	[54]

## Characterising the attachment of polyelectrolytes onto gold nanoparticles

Typically, the attachment of PE coatings on AuNPs is characterized using Surface Plasmon Resonance (SPR), Dynamic Light Scattering (DLS) and zeta potential. Other methods such as Transmission Electron Microscopy (TEM) and Nuclear Magnetic Resonance (NMR) are commonly used but will not be discussed here.

### Surface plasmon resonance

**Fig. 2:**
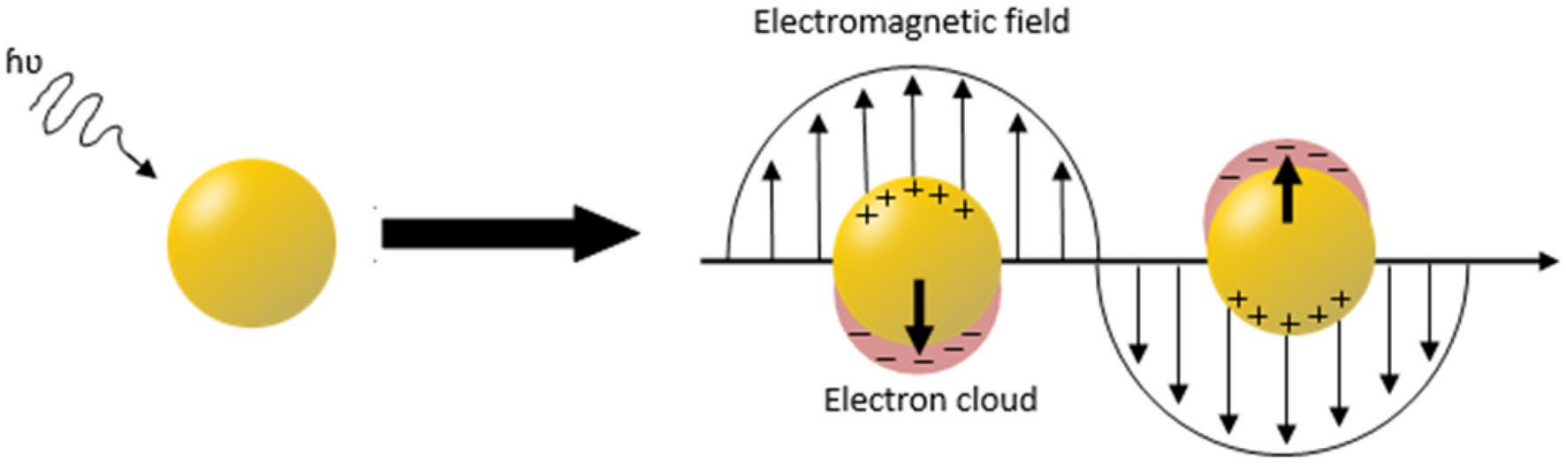
Schematic representation of the conduction electrons oscillating across the gold nanoparticle in the electromagnetic field of incident light

A surface plasmon is a charge-density oscillation phenomenon, which exists at the interface of two media with dielectric constants of opposite signs [55]. Nobel metals have a negative dielectric constant and are therefore ideal materials for surface plasmon detection. When light of a specific wavelength interacts with a AuNP, it causes a collective oscillation of the free electrons in the metal [56]. When the incoming electromagnetic wave has the same wavevector as the oscillating conduction electrons, resonance occurs (Fig. 2) and the incoming energy is absorbed into the plasmon wave. Typically, SPR is observed by measuring the absorbance of the NP-containing solution as a function of wavelength. The resulting signal depends on the shape, size, surface ligand, solvent, temperature and proximity of other NPs in the solution [57, 58]. For example, spherical AuNPs exhibit size dependent absorption peaks (surface plasmon band) from 500 to 550 nm [11, 59].

**Fig. 3:**
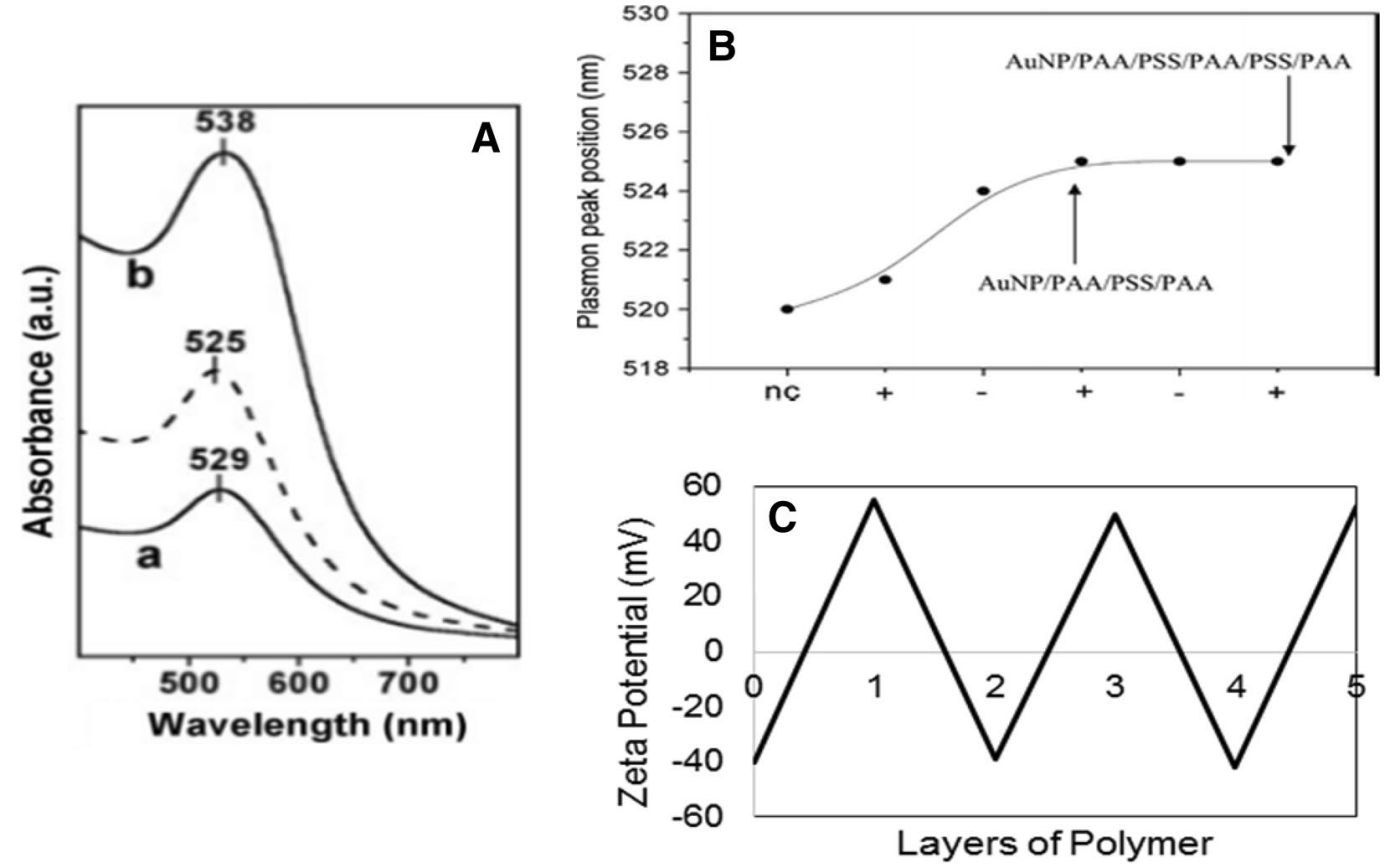
**A** UV–Vis spectra of mercaptoundecanoic acid coated AuNPs (dashed line) subsequently coated in (a) Polydiallyldimethylammonium chloride (PDADMAC) and (b) PDADMAC/polystyrenesulfonate (PSS). Reproduced from Ref. [61] with permission from Wiley. **B** Surface plasmon band shifts compared to the number of polyelectrolyte coatings on the AuNPs. Reproduced from Ref. [52] with permission from Wiley. **C** Zeta potential of uncoated citrate capped AuNPs (0) then subsequent addition of positively charged polyallylamine (PAA) and negatively charged polystyrenesulfonate (PSS). Reproduced and adapted from Ref. [52] with permission from Wiley

Aggregation of NPs can be observed by a red-shifting and broadening of the SPR peak, and the colour of the NP solution changes from a red to blue due to interparticle plasmon coupling [60]. The addition of a polymer layer onto the surface of the AuNP results in a change in the dielectric properties at the nanoparticle surface which shifts the SPR peak (Fig. 3a, b) [30]. As each polymer layer is added, the peak’s wavelength increases as the dielectric property changes. Due to this, the Lbl addition of PE multilayer architectures can be monitored through changes in the NPs SPR peak (Fig. 3a).

### Zeta potential

Measuring the zeta potential of AuNPs is a quick and efficient way to determine the charge of the particles and the stability of the colloidal system [62]. The zeta potential can be described as the potential at the shear plane of a solid particle moving under an electric field, where the shear plane is the boundary at the solid–liquid interface, between the stationary and diffuse layers [63, 64]. At neutral pH, citrate capped AuNPs have a resulting negative charge, which attracts positively charged electrolyte ions. These ions then form an electrical layer at the surface of the particle, known as the Stern layer. A secondary layer called the diffuse layer also forms, which consists of both positive and negative ions with a high counter-ion charge. Together these two layers make up an electrical double layer. The zeta potential of the particle corresponds to the amount of energy required to shear a particle and associated double layer from the bulk solution. In order to calculate the zeta potential, the electrophoretic mobility of a particle is measured in a direct current electric field [62]. Stable colloidal solutions typically have zeta potentials smaller than − 30 mV or larger than + 30 mV. Values between these potentials indicate unstable solutions, as the particles do not carry enough charge to repel each other, which can lead to aggregation [65]. In the case of PE-coated NPs, the charge of the coated particles alternates between a positive and negative charge, depending on the polymers used (Fig. 3c). This process is often used to show attachment of the polyelectrolyte layer, as well as to confirm the colloidal stability of the system.

## Uptake and interaction with cells

Biocompatibility is essential in any biomedical application as well as the ability for the uptake of NPs into cells, especially for applications such as drug delivery. The interactions of the particle with the cell membrane is ultimately what determines cellular uptake, as it is the key to the regulation of the uptake process [66]. Cellular interactions and cellular uptake is a large and complex topic area and hence, this section will only discuss the main considerations with select examples. More in-depth explanations in this area can be found in other review articles [67, 68]. The cellular uptake of AuNPs is dependent on the size, charge and surface properties of the NP [69]. Other biological factors include the type of cell, cellular recognition and the temperature [70]. These biological factors also affect the protein corona, which is a protein layer attached to the AuNP, brought about by the proteins found in the body (in vivo*)* and in serum (in vitro*)* which attach to the NPs surface [70].

There are five ways in which mammalian cells can internalize nanoparticles: phagocytosis, macropinocytosis, clatherin-mediated, caveolin-mediated and clatherin/cavoelin-independent endocytosis [71–74]. Of these pathways, clatherin and caveolin mediated endocytosis are often widely grouped as receptor mediated endocytosis [66, 75–77]. It has been proposed that receptor mediated endocytosis is the primary mechanism for cellular entry of AuNPs less than 100 nm in diameter into mammalian cells [67, 78]. In receptor mediated endocytosis, NPs which have ligands on their surface that target specific receptors, attach to the cells by receptor–ligand binding. The membrane of the cell then wraps around the NP, internalizing it into the cell. The kinetics of this process depend on the size of the NP, with generally a faster uptake for larger NPs [70, 78]. However, the size and uptake will change depending on the protein corona and its composition when it forms around the particle. The addition of serum to in vitro essays provides a more realistic biological environment, as the proteins in the serum can attach to the NP forming the protein corona. This corona prevents the NP from having direct contact with the cell membrane, hence altering the uptake. The uptake rate is determined by the receptor diffusion kinetics and the thermodynamic driving force for the membrane wrapping [78]. This rate was shown to be dependent on the cell type, particle size and the composition of the protein corona.

Fastest cell uptake has been shown for NPs with diameters of around 55 nm. The chemical energy released by the receptor–ligand interaction produces enough free energy (thermodynamic driving force) to drive the NP into the cell [78–80]. For NPs smaller than 40 nm in diameter, the receptor–ligand interactions cannot provide enough energy to ‘wrap’ the NP on the cell’s surface as there are fewer ligands that can interact with receptors, and not enough chemical energy is produced to overcome the more unfavorable deformation of the cell membrane [80]. In order for smaller NPs to be taken into the cell, clusters of NPs are required to overcome the energy barrier to internalization [78]. However, when taking into account the protein corona, for 50 nm diameter AuNP in serum, the presence of the corona decreased the uptake efficiency significantly, with a 70% decrease in RAW 264.7 cells (model mouse macrophage cells) and a 40% decrease in Hep G2 cells (human liver cancer cells). For 20 nm and 5 nm AuNP, it was found the inhibitory effect of the corona on cellular uptake becomes negligible for both types of cells [70]. Even with a considerably lower uptake efficiency, the uptake was still higher in larger (50 nm) AuNP, suggesting for biomedical applications, ≥ 50 nm AuNPs should be used [70].

The influence of the surface charge on cellular uptake is relatively well understood, but also dependant on a number of experimental factors. These experimental factors, including particle size, surface functionalisation, NP shape and cell type are often observed to interplay with each other, making identifying differences of one variable a complex endeavour. Typically, positively charged NPs are more easily taken up by the cell, probably due to being attracted by the negatively charged cell membrane [81–83]. However, proteins from the growth serum of cells can absorb to both cationic and anionic particles, forming a protein corona which has the potential to alter their charge and thus minimizes attractive forces [84, 85]. The effect of proteins adsorbing onto AuNPs has been observed through numerous studies including absorbtion on curcumin-functionalized AuNP where the uptake in human prostate cancer cells was decreased when curcumin-AuNP were in the presence of serum containing media compared to serum-free media [86].

The localization of the NP inside the cells is important for biomedical applications but once again the intracellular distribution of AuNPs depends on a number of factors including size, concentration, and serum/media type. NPs with no specific surface functionalisation and a diameter of less than 6 nm have been shown to enter the nucleus of various cells [87–89]. Similarly, other studies have observed that diameters of greater than 6 nm do not enter the nucleus but often enter cells inside vesicles [78, 90–92]. Knowing the size range of NPs which reach the nucleus is important for inducing apoptosis in cells when treating conditions such as cancer and therefore, needs to be taken into consideration depending on the application.

The biodistribution of AuNP when injected into mice, was observed to change depending on the charge of a 2 nm core AuNP [93]. Although the research did not use PEs to alter the charge, similar results would be expected when using PEs. It was found that positively charged NPs accumulate in the filtering regions of the spleen and liver, indicating they filter from the bloodstream at a faster rate than anionic or neutral NPs. The neutral NPs were shown to accumulate in the arteries and negatively charged NPs were found homogenously distributed within the kidney. Another key difference was the neutral NPs which were shown to interact with the immune system. This interaction however, may be due to the type of proteins which make up the corona surrounding the particle [93].

In terms of PE coated AuNP, there was very little research found surrounding the uptake and cellular interactions. Thus, the majority of uptake and interaction research has come from studies on AuNP with a large variety of coatings. When comparing experiments for AuNPs, despite it being such a large research area, it is difficult to form conclusions for in vitro studies, as there are many complex parameters which dictate the cellular response. The cell type, serum used, AuNP size, concentration and charge among others can alter the outcome significantly. Similarly another difficulty is being able to predict the biodistribution as the protein corona which forms when the NPs enter the body, (i.e. blood, lung etc.) changes as the NP is transported through different regions such as the bloodstream [93]. Overall the uptake and distribution is a complex interplay between different experimental factors which makes drawing direct comparisons difficult.

## Applications

The unique optical and physical properties of AuNPs and the increased biocompatibility of PE-coated nanoparticles has led to a range of potential biomedical applications, including drug delivery, gene therapy and cancer therapy (Table 1) [50, 52, 53, 69, 94]. The type of PE used has a significant influence on the usage of particles and the interaction of particles with tissues and cells. A fundamental understanding of the influence of the PEs on interactions with biological material is hence crucial to optimize their use. There are two main delivery methods for drug delivery: targeted and non-targeted. In targeted delivery, a ligand, generally an antibody or peptide, is attached to the NP and will target a receptor on a specific cell [95, 96]. Non-targeted delivery is the delivery without targeting specific cells, where the drug will be released to both healthy and diseased cells. The type of delivery is extremely important in applications such as gene therapy and photothermal therapy, where treatment needs to be given into a specific area of the body. siRNA delivery for example will target a specific cell’s cytoplasm to achieve down-regulation (80–90% decrease) of an overproduced protein [97]. Similarly in photothermal therapy, ensuring the AuNPs reaches the diseased cells to cause cell death rather than healthy cells is vital to reduce collateral damage [98].

### Drug delivery

Targeted drug delivery allows for an increased concentration of drug to be given to a specific cell type compared to conventional untargeted delivery. In many cases this higher concentration increases the efficacy and decreases unwanted side effects, especially useful for drugs that can have debilitating side effects such those used in chemotherapy. Attaching ligands such as antibodies to the surface of a drug carrier allows drug release into specific cells which have the correct receptors.

**Fig. 4:**
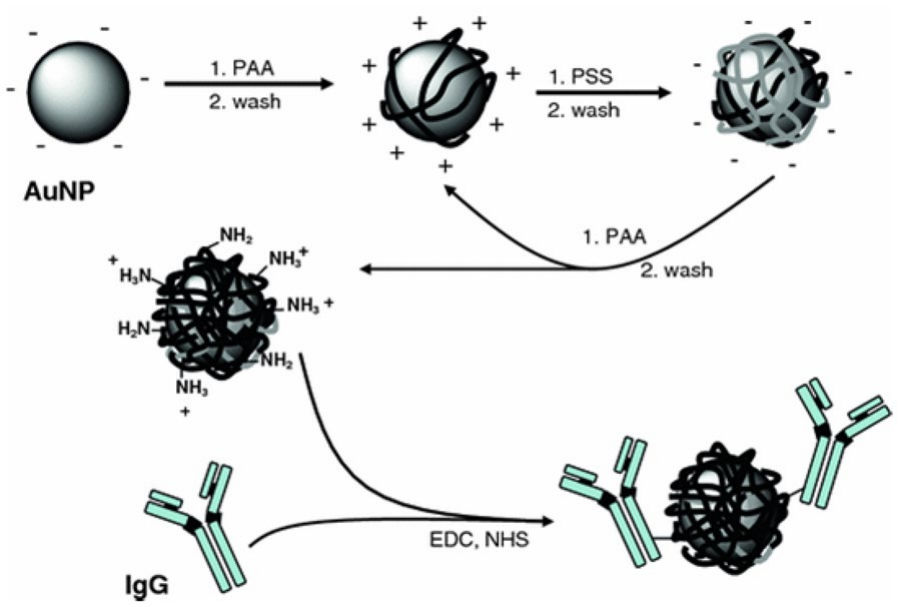
Citrate stabilized gold nanoparticles coated with PSS and PAA in a Lbl method with the addition of IgG antibody through amide linkages. Reproduced from Ref. [52] with permission from Wiley

For targeted drug delivery, PE coatings can be used as anchoring points for antibodies. This was shown in a proof of concept study, where IgG monoclonal antibodies, which target proteins overexpressed in cancer cells, were anchored to the surface of polyallylamine (PAA) and PSS coated AuNPs (Fig. 4) [52]. Conceptually, this could have great implications for the cancer treatments, as drugs could be loaded inside the AuNP or within the PE layers and then be targeted to cancer cells by the antibody attached on its surface. This would mean a lower dose of chemotherapy could be given with less systemic exposure, lessening the side effects and increasing the survival rate. Issues, however, with this type of antibody targeting is the administration into the body, where unlike an in vitro test there are many proteins which attach themselves to foreign bodies like NPs, making the receptor to antibody attachment problematic.

One of the advantages of the PE systems is that water insoluble drugs, which previously have been difficult to deliver, now have an easier pathway [99]. This is shown to be possible through the use of multilayer based drug carrier systems, where a vehicle such as a NPs can have water-insoluble drugs encapsulated within its PE chains. In a proof of concept study, three polymer layers with one containing a water-insoluble drug, were absorbed onto a NP carrier and this carrier was able to increase the drug deposition efficiency by a factor of 100 [53]. In order to trap the drug, citrate coated AuNPs were used, then subsequently coated with polyallylamine hydrochloride (PAH) before the complexed drug was mixed with PSS and electrostatically attached to the PAH coating.

**Fig. 5:**
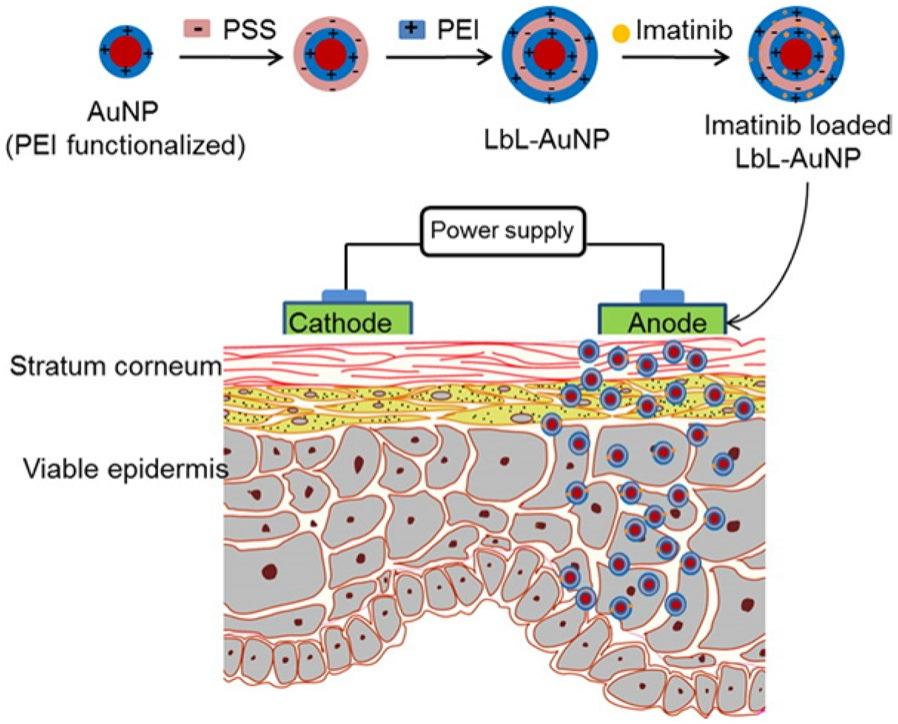
A schematic of the cancer drug, Imatinib Mesylate being encapsulated in the multilayer coated PEI/PSS/PEI AuNPs, showing the use of iontopherisis for topical delivery into the layers of the skin. Reprinted from Ref. [48] with permission from the American Chemical Society

A more recent proof of concept study has found that the drug, Imatinib Mesylate (IM), used for cancer treatment could be encapsulated into PSS/PEI multilayer functionalized gold nanoparticles (Fig. 5). This IM-PSS/PEI-AuNP system was tested in several ways with uptake into B16F10 murine melanoma cells measured as well as an in vitro skin penetration study [48]. At a gold concentration greater than 50 μM and IM concentrations above 31 μM, the IM-PSS/PEI-AuNP had a significantly higher growth inhibition of cancer cells compared to IM alone. The in vitro skin penetration studies conducted using pig ear skin, showed the use of iontopherisis (voltage gradient on the skin) enhanced the skin penetration of the IM-PSS/PEI-AuNPs. Thus a topical treatment of IM-PSS/PEI-AuNPs with iontopheresis shows potential for enhanced melanoma treatement compared to IM alone.

### Gene therapy

Small interfering RNA (siRNA) delivery has the potential to be used in therapeutics to temporarily silence genes which could have significant effects on genetic diseases, however siRNA are notoriously difficult to deliver due to instability [100]. PE coated AuNPs have been shown to be better delivery vehicles than commonly used polymer vehicles [49]. Other delivery platforms such as cationic lipids and polymers have shown promise however instability is often the biggest issue, leading to decreases in efficacy [69]. In a proof of concept study, siRNA has been attached onto a PE coated AuNP system and delivered into CHO-K1 cells (hamster ovary cells) which express enhanced green fluorescent protein (EGFP) [69]. The siRNA successfully reduced the EGFP expression and the cells remained viable after the addition of the AuNPs.

The current gold standard for non-viral gene transfection is using PEI or Lipofectamine. PEI is a polyelectrolyte in its own right, however, in this case, it is used to form a polyplex with DNA, rather than as a coating. This gold standard has been recently challenged in a study which synthesized PEI (25 kDa) coated AuNP as DNA nanocarriers which found that these nanoparticles were more efficient gene vectors than both Lipofectamine and un-modified PEI [49]. PEI coated nanoparticles also showed low cell cytotoxicity and were fabricated in a simple one-pot method. Thus PE coated gold nanoparticles show great promise for gene transfection in the future, as they can out-perform the current gold standard.

One of the challenges with using PEs is that the strong interaction of oppositely charged polymers often leads to retardation of the payload release. Changing the pH of the PE system can be a way to overcome this problem by allowing for a charge reversal of the PE in acidic conditions, leading to release of the siRNA. Using the charge reversal method, a study which used PEI/PAH-Cit/PEI/MUA-AuNPs to release siRNA into cancer cells was able to knock-down 80% of Lamin A/C protein expression in acidic conditions, whereas the siRNA remained attached at a neutral or basic pH [94]. Interestingly, the siRNA was released with 14% more efficiency than what is commercially available for this knockdown.

### Photothermal therapy

Hyperthermia is a cancer treatment that has been used since the early 1990s [101]. When heat, typically just above the physiological temperature, is generated in a region of the body, it can lead to damage and destruction of cells [101]. In Photothermal therapy, the heating is more intense and is applied to a specific area though the use of NPs, resulting in fewer unwanted side effects compared to the hyperthermic treatment [102]. AuNPs can be targeted to a site in the body (i.e. a tumor) by manual injection or through targeted delivery. An external laser with a wavelength between 650 and1350 nm is then aimed at the specific site where the NPs absorb or scatter that light [103]. This wavelength range is important as it can deeply penetrate through healthy tissue to reach the AuNPs. The absorbed light causes resonance and is converted into heat, which is released to the surrounding tissue causing cellular death. Although this concept has been shown in several examples, so far PE coated AuNPs have not been used. However, PE-coated GNRs have been shown to be effective in killing cancer cells in mice and cell lines through photothermal therapy [104–107]. Compared to GNR, there is little research surrounding spherical AuNPs for use in Photothermal therapy, likely due to the potentially limited applications, as this therapy seeks to penetrate and heat the deeper tissue with near infrared (NIR) light. As spherical nanoparticles tend to have absorbance between 500 and 550 nm, this does not target the NIR region and thus will not penetrate deep tissue in the same way as other shapes such as nanorods would [108]. Recent studies using AuNP have overcome this by using aggregated or clustered nanoparticles, as this shifts the absorbance peak to higher wavelengths, allowing the wavelength of absorption to be within the therapeutic window [109, 110].

### Imaging

Imaging in medicine is an important tool for a number of procedures including the localization and diagnosis of cancers. The optical properties of gold make it very attractive for use as a contrast agent in imaging. So far, PE coated GNRs have been used for imaging applications rather than spherical AuNPs. A comprehensive review on PE GNRs and their imaging capabilities can be found by Pissuwan and Niidome and is not within the scope of this review [29].

Photoacoustic (PA) imaging and computed tomography (CT) are also useful tools in clinical practice for imaging. PA imaging is based on the PA effect, where pulsed laser light is utilized as probing energy which produces acoustic waves by thermal expansion. These waves are then detected at the surface of the tissue by ultrasound and are re-constructed to form an image [111]. A study in which PEG-b-poly(ɛ-caprolactone) was tethered to AuNPs showed a strong plasmon coupling effect where NIR absorption induced plasmon coupling causing an increase in PA signal with high conversion efficiency [112]. Similarly AuNP have also shown promise as CT contrast agents due to their favourable properties. CT images are produced by a combination of X-ray images taken at different angles by rotation around an object, to form a cross-sectional 3D image known as a CT scan. Depending on what is being imaged, contrast agents can be used to highlight specific areas such as blood vessels or the tissue structure of organs by attenuating the X-rays to improve image quality [113]. Gold nanoparticles are being explored for their use as a contrast agent in CT due to gold having a high atomic number and electron density, meaning it has good X-ray attenuation ability. In an experiment using a range of sizes of PEGylated AuNP as a contrast agent for CT scans, it was found NPs with a size of 13.2 nm and sizes greater than 34.8 nm performed ~ 20% better in attenuation intensity than Idohexal, a common CT contrast agent [114]. Thus the area of imaging is extremely promising for PE coated AuNP and AuNPs in general.

## Toxicity

The cytotoxicity of AuNPs is extremely important, especially if they are being used in the biomedical field. The cytotoxicity is dependent on the size, shape, functionalisation, surface charge and aggregation of the NPs as well as biological factors including the type of cell and the uptake mechanisms into the cell [115–119]. PE coatings on AuNPs appear to be relatively non-toxic to cells, however other factors including the type of coating and the NP size need to be considered. For example, PAH coated 18 nm diameter AuNP were compared with CTAB, citrate and poly(acrylic acid) (PAA) coated AuNPs. They were exposed to SH-SY5Y (human neuroblastoma) cells for 24 h and after incubation all coatings except the CTAB coating showed ˃ 95% cell viability. Interestingly, when 40 nm PAA coated AuNP were used, cell viability dropped considerably to only 10% using the same gold concentration [120]. Another study has a similar result, where 10 nm diameter PEI coated AuNP showed to be biocompatible through in vitro cytotoxicity studies, where in gold concentrations of up to 400 μM they were shown to be non-toxic to three different cancer cell lines (HCT116 colorectal carcinoma, MCF7 breast adenocarcinoma and PC3 prostate adenocarcinoma) [121]. Whereas, the 23 nm diameter PEI coated AuNP showed a moderate level of cytotoxicity to the same three cancer cell lines, with IC_50_ results below 80 μM [121]. In both experiments the larger nanoparticles proved to be more cytotoxic then their smaller counterparts. In contrast to this, a recent study has found that in vitro toxicity of PEG coated AuNPs is dependent upon size of the particles and the dose, with smaller size and higher concentration leading to increased cytotoxicity [2]. Similarly in another study, 1.4 nm AuNPs were found to be 100 times more toxic than 15 nm AuNPs using the same coating [122]. Thus there are discrepancies found about size and toxicity throughout the literature which further emphasizes the complexity of experimental design, especially when testing in vitro.

With many discrepancies found, drawing conclusions from the collective body of evidence is difficult as results vary depending on a number of experimental factors. The toxicity seems to be dependent on the size, surface functionalisation, concentration and surface charge of the AuNP as well as cell type, serum used, incubation time etc. Although there are many studies on AuNP toxicity, there are very few studies on the toxicity of PE coated AuNP. In order to understand the role PEs play in the toxicity space, standardized protocols for both in vivo and in vitro studies including appropriate cell types, assays and dosages would be beneficial to directly compare different coatings to determine the toxicity in a range of cell types.

## Conclusions and future outlook

The available literature on PE coated spherical AuNPs is limited compared to AuNP with other coatings or its PE coated GNR counterpart. The interaction of AuNP with cells as well as their uptake is dictated by a range of parameters and despite a large body of research, drawing conclusions is difficult as there are many inconsistencies and complexities to consider. Variables such as the cell type, the medium used, AuNP size, concentration and charge, among others, can significantly alter the outcome and hence care needs to be taken when comparing results from literature.

In this review it was found that typically, ≥ 50 nm diameter AuNP have the highest uptake efficiency compared to smaller AuNPs. In terms of biomedical applications, PE coated AuNPs have shown great potential in areas such as SiRNA delivery, imaging and drug delivery. PE coated AuNPs have shown to be very effective as siRNA carriers—even more so than the current gold standard of gene transfection. In addition, proof-of-concept studies have indicated they can be used as effective drug delivery vehicles. Also the use of AuNP in imaging techniques such as photoacoustic imaging and computed tomography have shown great promise in initial studies, with PEGylated AuNPs out-performing Idohexal, a common CT contrast agent.

There is however, a large gap of knowledge and more research is required to determine how various experimental parameters (size of the PE, salt concentration of PE mixture, strength of the attached anion on the AuNPs surface and pH) play a role in the deposition of PEs onto the AuNPs. Other challenges include how the addition of the PE coatings on AuNPs affects the cellular uptake and interactions with cells both in vitro and in vivo. A standardized protocol for both in vivo and in vitro studies would be beneficial to directly compare different coatings and to determine the toxicity in a range of cell types. The future outlook for the use of PE coated AuNPs is positive, and this review has shown several of the possible uses in biomedicine. There is still much research to be conducted to better understand these systems and how each individual parameter impacts how they interact with cells but the initial literature in this area shows promise that PE coated AuNPs could reach their potential in the biomedical field.
